# Expression of inwardly rectifying potassium channels (GIRKs) and beta-adrenergic regulation of breast cancer cell lines

**DOI:** 10.1186/1471-2407-4-93

**Published:** 2004-12-16

**Authors:** Howard K Plummer, Qiang Yu, Yavuz Cakir, Hildegard M Schuller

**Affiliations:** 1 Molecular Cancer Analysis Laboratory, Department of Pathobiology, College of Veterinary Medicine, University of Tennessee, Knoxville, TN 37996-4542, USA; 2 Experimental Oncology Laboratory, Department of Pathobiology, College of Veterinary Medicine, University of Tennessee, Knoxville, TN 37996-4542, USA; 3 Current address: Department of Pathology, University of Alabama at Birmingham, Birmingham, AL 35294-0019, USA

## Abstract

**Background:**

Previous research has indicated that at various organ sites there is a subset of adenocarcinomas that is regulated by beta-adrenergic and arachidonic acid-mediated signal transduction pathways. We wished to determine if this regulation exists in breast adenocarcinomas. Expression of mRNA that encodes a G-protein coupled inwardly rectifying potassium channel (GIRK1) has been shown in tissue samples from approximately 40% of primary human breast cancers. Previously, GIRK channels have been associated with beta-adrenergic signaling.

**Methods:**

Breast cancer cell lines were screened for GIRK channels by RT-PCR. Cell cultures of breast cancer cells were treated with beta-adrenergic agonists and antagonists, and changes in gene expression were determined by both relative competitive and real time PCR. Potassium flux was determined by flow cytometry and cell signaling was determined by western blotting.

**Results:**

Breast cancer cell lines MCF-7, MDA-MB-361 MDA-MB 453, and ZR-75-1 expressed mRNA for the GIRK1 channel, while MDA-MB-468 and MDA-MB-435S did not. GIRK4 was expressed in all six breast cancer cell lines, and GIRK2 was expressed in all but ZR-75-1 and MDA-MB-435. Exposure of MDA-MB-453 cells for 6 days to the beta-blocker propranolol (1 μM) increased the GIRK1 mRNA levels and decreased beta_2_-adrenergic mRNA levels, while treatment for 30 minutes daily for 7 days had no effect. Exposure to a beta-adrenergic agonist and antagonist for 24 hours had no effect on gene expression. The beta adrenergic agonist, formoterol hemifumarate, led to increases in K^+ ^flux into MDA-MB-453 cells, and this increase was inhibited by the GIRK channel inhibitor clozapine. The tobacco carcinogen 4-(methylnitrosamino)-1-(3-pyridyl)-1-butanone (NNK), a high affinity agonist for beta-adrenergic receptors stimulated activation of Erk 1/2 in MDA-MB-453 cells.

**Conclusions:**

Our data suggests β-adrenergic receptors and GIRK channels may play a role in breast cancer.

## Background

Breast cancer is the leading cancer in women [1] and estrogen receptor (ER)(-) breast cancers have a poorer prognosis than ER(+) cancers [2,3]. Smoking is a controversial risk factor for the development of these malignancies [4-7]. However, increases in pulmonary metastatic disease and lung cancer have been seen in smokers with breast cancer [8,9]. The tobacco-specific nitrosamine 4-(methylnitrosamino)-1-(3-pyridyl)-1-butanone (NNK) causes cancer of the oral cavity, esophagus, respiratory tract and pancreas, but no breast cancer in laboratory animals [10] and has not been implicated in breast carcinogenesis to date.

Recent studies in human cancer cell lines or in animal models have shown that the growth of adenocarcinomas of the lungs, pancreas and colon are under β-adrenergic control [11-15]. Studies in a cohort of 2442 men found an inverse association between risk of incident adenocarcinomas of the prostate and use of antihypertensive medication, including beta-blockers [16]. The tobacco-specific carcinogenic nitrosamine NNK has recently been identified as a high affinity β-adrenergic agonist that stimulated the growth of pulmonary and pancreatic adenocarcinomas in vitro and in animal models [11,13,15]. The expression of β-adrenergic receptors has been correlated with the over-expression of the arachidonic acid-metabolizing enzymes cyclooxygenase-2 (COX-2) and lipoxygenases (LOX) in adenocarcinomas of lungs [17], colon [18], prostate [19], and pancreas [15]. Inhibitors of these enzymes have been identified as cancer preventive agents in animal models of these cancers [13,20-22]. Collectively, these findings suggest that among the superfamily of adenocarcinomas at various organ sites, there is a subset of malignancies that is regulated by β-adrenergic and arachidonic acid-mediated signal transduction pathways.

The majority of breast cancers are also adenocarcinomas and many of them over express COX-2 and/or LOX [23]. This raises the possibility that comparable to findings in adenocarcinomas of the lungs, pancreas, colon and prostate, a subset of breast cancers may also be under beta-adrenergic control. In support of this hypothesis, studies have demonstrated that three estrogen-responsive and three non-estrogen responsive human cell lines derived from breast adenocarcinomas demonstrated a significant reduction in DNA synthesis in response to beta-blockers or inhibitors of the arachidonic acid-metabolizing enzymes COX-2 and 5-LOX [24]. In addition, analysis by reverse transcription polymerase chain reaction (RT-PCR) revealed expression of β_2_-adrenergic receptors in all six breast cancer cell lines tested (MDA-MB-361, ZR-75-1, MCF-7, MDA-MB-453, MDA-MB-468, MDA-MB-435S), whereas β_1 _receptors were not found in two estrogen non-responsive cell lines (MDA-MB-435S, MDA-MB-453) [24].

Expression of mRNA that encodes a G-protein coupled inwardly rectifying potassium channel (GIRK1) has been shown in tissue samples from approximately 40% of primary human breast cancers tested [25], and this expression of GIRK1 was associated with a more aggressive clinical behavior. Increases in GIRK currents by beta-adrenergic stimulation have been reported in adult rat cardiomyocytes and in Xenopus laevis oocytes coexpressing β_2_-adrenergic receptors and GIRK1/GIRK4 subunits [26]. In addition, in rat atrial myocytes transiently transfected with β_1 _or β_2 _adrenergic receptors, the beta-adrenergic agonist isoproterenol stimulated GIRK currents, whereas this stimulation was not seen in non-transfected cells [27]. The current investigations test the hypothesis that GIRK1 channels in human breast cancers are correlated with beta-adrenergic control.

## Methods

### Cell culture

The ER(+) human breast cancer cell lines MDA-MB-361, ZR-75-1, and MCF-7 and the ER(-) cell lines MDA-MB-453, MDA-MB-468 and MDA-MB-435S were purchased from the American Type Culture Collection (Rockville, MD). Cells were maintained in RPMI 1640 medium supplemented with fetal bovine serum (10%, v/v), L-glutamine (2 mM), 100 U/ml of penicillin and 100 μg/ml streptomycin (Invitrogen-Life Technologies, Grand Island, NY) in an environment of 5% CO_2_. Exposure of cells to propranolol, isoproterenol, or clozapine (Sigma, St. Louis, MO), NNK (Chemsyn, Lexena, KS), or formoterol hemifumarate (Tocris, Ballwin, MO) for experiments was as detailed in the Figure Legends.

### RT-PCR

RNA was isolated by Trizol reagent (Invitrogen-Life Technologies) or by an Absolutely RNA kit (Stratagene, La Jolla, CA). RT-PCR was done as previously described [28]. The GIRK1 primers are forward 5'-ctatggctaccgatacatcacag-3' and reverse 5'-ctgttcagtttgcatgcttcgc-3' which span exon 1 and 2 [29] and amplifies a 441 bp fragment (bases 631–1072, Genbank Acession # NM_002239). The GIRK2 primers are forward 5'-atggatcaggacgtcgaaag-3' and reverse 5'-atctgtgatgacccggtagc-3' amplifies a 438 bp fragment (bases 700–1137, Genbank Acession #U52153). The GIRK4 primers are forward 5'-aaccaggacatggagattgg-3' and reverse 5'-gagaacaggaaagcggacac-3' which amplifies a 401 bp fragment (bases 117–517, Genbank Acession # L47208). PCR conditions are 94°C, 30 sec; 55°C, 30 sec; 72°C, 45 sec for 40 cycles. Cyclophylin primers were used as an internal control (Ambion, Austin, TX).

### Relative competitive RT-PCR

Preliminary experiments were done with MDA-MB-453 cells to determine a cycle number of PCR amplification that is within the linear range, which is critical for meaningful results to compare expression levels between samples and to determine the mixture of 18S primers/18S competimers (Ambion-Classic II). The 18S ribosomal RNA primers/competimers are used as an invariant internal control, which allows correction for sample variation. Results indicated this was 31 cycles of PCR and a 1:9 18S primer/competimers ratio. For experimental treatments, as described before [33], cDNA was made and PCR performed except reactions were spiked with 5 μCi [α-^32^P]-dCTP (3000 Ci/mmole, Dupont-NEN, Boston, MA). Reactions were run with the following conditions: 1 cycle of 2 min. at 94°C, then 31 cycles of 94°C, 30 sec; 55°C, 30 sec; 72°C, 45 sec. A 10 μl sample of each PCR reaction was heated at 95°C for 3 min., then loaded into a 5% TBE-urea Ready Gel (Bio-Rad, Hercules, CA). This underwent electrophoresis at 200 V in TBE buffer until the xylene cyanol dye front reached the bottom of the gel. The gel was transferred to filter paper, dried and exposed to film or imaged on a Molecular Dynamics 445 SI phosphoimager (Sunnyvale, CA). A 100 bp DNA ladder (Invitrogen-Life Technologies) was exchange labeled with T4 polynucleotide kinase and 30 μCi [γ-^32^P] ATP (3000 Ci/mM, Dupont-NEN).

### Real-time PCR

The GIRK-1 primers for real time PCR are forward 5'-ctctcggacctcttcaccac-3' and reverse 5'-gccacggtgtaggtgagaat-3' (bases 398–477, Genbank Acession # NM002239). and the internal TaqMan probe is 6-FAM-tcaagtggcgctggaacctc-TAMRA (bases 429–449, Sigma-Genosys, The Woodlands, TX), annealing temperature 62°. GIRK2 primers-forward 5'-gacctgccaagacacatcag-3' and reverse 5'-cggtcaggtagcgataggtc-3' (bases 766–886, Genbank Acession # U52153) and the internal TaqMan probe is 6-FAM-gtgcaatgttcatcacggcaac-TAMRA (bases 837–859), annealing temperature 56°. GIRK4 primers-forward 5'-agcgctacatggagaagagc-3' and reverse 5'-aagttgaagcgccacttgag-3' (bases 241–358, Genbank Acession # L47208) and the internal TaqMan probe is 6-FAM-accggtacctgagtgacctcttca-TAMRA (bases 301–324), annealing temperature 62°. Reactions were run on a Cepheid SmartCycler (Sunnyvale, CA). Reaction conditions are 200 μM dNTPs, 0.3 μM gene specific primers, 0.2 μM TaqMan probe, 4 mM (GIRK1) or 6 mM (GIRK2or4) magnesium acetate, 2 μl cDNA and 1.5 U MasterTaq (Eppendorf, Westbury, NY) and MasterTaq buffer in a final volume of 25 μl. TaqMan beta-actin detection reagents (Applied Biosystems) were used with the same reaction conditions as above except a 5 mM magnesium concentration was used and this was run at 95° for 120 seconds, followed by 45 cycles of 95°, 15 seconds; 68°, 30 seconds.

### Measurement of potassium flux

We determined inward potassium flux in these cells by flow cytometry via the method of Krjukova et al. [30]. The negatively charged fluorescent dye bis-(1,3-dibutylbarbituric acid)trimethine oxonol (DiBaC_4_(3)) (Molecular Probes, Eugene, OR) was added to MDA-MB-453 breast cancer cell line suspensions of 1 × 10^6 ^cells at a final concentration of 150 × 10^-9 ^M. Fluorescence intensity measurement after treatment of the cells was obtained from a FACS Vantage/SE Cell Sorter (San Jose, CA).

### Analysis of protein expression by western blots

Following incubation with agents as detailed in the Figure legends, cells were washed twice with phosphate buffered saline and lysed with cold RIPA lysis buffer containing protease inhibitors (50 mM Tris pH 7.4, 150 mM NaCl, 1% NP-40, 1% Triton × 100, 0.1% SDS, 1% sodium deoxycholate, 1 mM EDTA, 50 mM NaF, 10 mM sodium pyrophosphate, 0.5 mM DTT). Cell lysates were collected from culture plates using a rubber policeman, and protein collected by centrifugation. Protein concentrations were determined by BCA protein assay (Pierce, Rockford, IL). Aliquots of 20 μg protein were boiled in 2x loading buffer (0.1 M Tris-Cl, pH 6.8, 4% SDS, 0.2% Bromophenyl blue, 20% glycerol) for 4 minutes, then loaded onto 10% Tris-HCl-Polyacrylamide gels (Biorad, Hercules, CA), and transferred electrophoretically to nictrocellulose membranes. Membranes were incubated with primary antibodies (phospho-Erk; Cell Signaling, Beverly, MA) and appropriate secondary antibodies (Cell Signaling or Rockland, Gilbertsville, PA or Molecular Probes, Eugene OR). In all western blots, membranes were additionally probed with an antibody for actin (Sigma) to ensure equal loading of protein between samples. The antibody-protein complexes were detected as previous described [28] or by the LiCor Odyssey infrared imaging system (Lincoln, NE).

## Results

**Figure 1:**
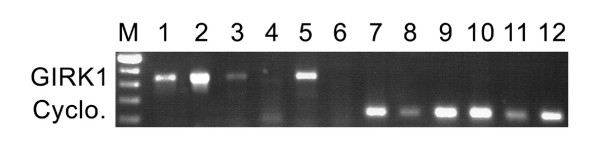
**Agarose gel showing expression of mRNA for GIRK1 in human breast cancer cell lines by RT-PCR**. The GIRK1 primers amplified a 441-bp fragment whereas the cyclophylin primers amplified a 216 bp fragment. For each cell line, a negative control reaction without M-MLV reverse transcriptase was performed and found to be negative. Lanes 1 & 7, ZR-75-1; Lanes 2 & 8, MCF-7; Lanes 3 & 9, MDA-MB-361; Lanes 4 & 10, MDA-MB-435S; Lanes 5 & 11, MDA-MB-453; Lanes 6 & 12, MDA-MB-468, Lane M, a 100 bp marker. PCR reactions resolved on this gel were in the plateau phase of PCR, therefore concentrations of PCR amplified cDNA samples cannot be compared.

**Figure 2:**
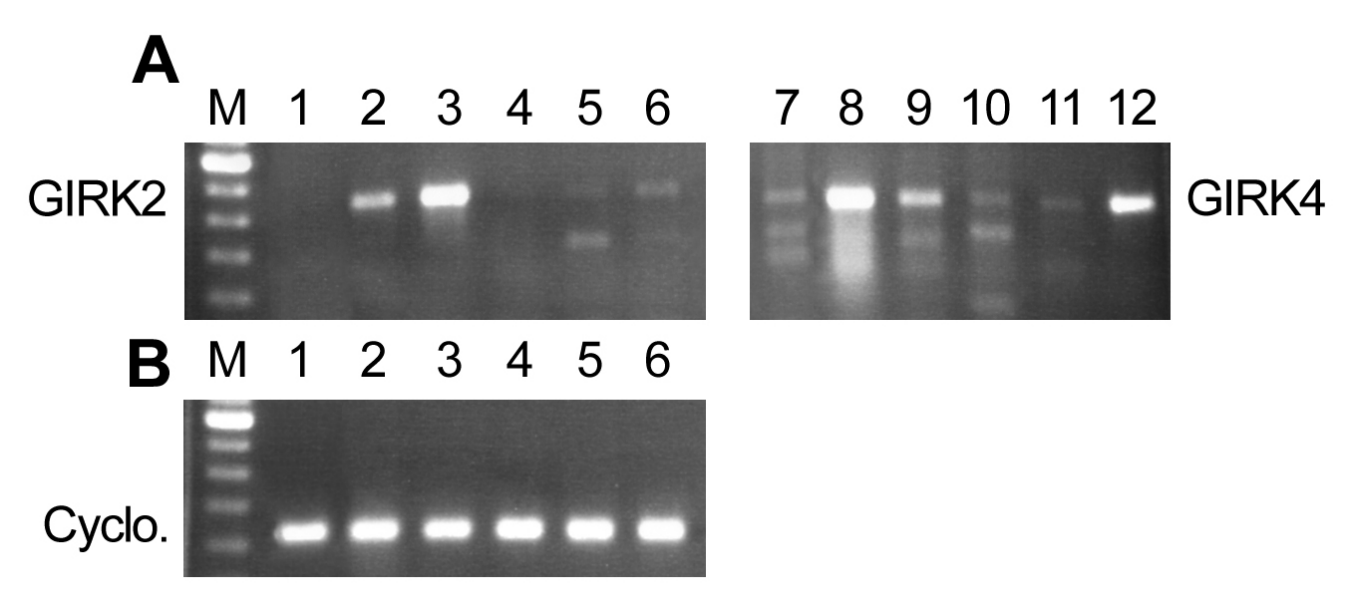
**Agarose gel showing expression of mRNA for GIRK2 and GIRK4 in human breast cancer cell lines by RT-PCR**. The GIRK2 and 4 primers amplified 438 & 401-bp fragments respectively, whereas the cyclophylin primers amplified a 216 bp fragment. Cyclophylin was used as a positive control for both GIRK2 and 4. For each cell line, a negative control reaction without M-MLV reverse transcriptase was performed and found to be negative. Lanes 1 & 7, ZR-75-1; Lanes 2 & 8, MCF-7; Lanes 3 & 9, MDA-MB-361; Lanes 4 & 10, MDA-MB-435S; Lanes 5 & 11, MDA-MB-453; Lanes 6 & 12, MDA-MB-468, Lane M, a 100 bp marker. PCR reactions resolved on this gel were in the plateau phase of PCR, therefore concentrations of PCR amplified cDNA samples cannot be compared.

The estrogen-responsive (MCF-7, ZR-75-1, MDA-MB-361) and estrogen non-responsive (MDA-MB-453, MDA-MB-435S, MDA-MB-468) human breast cancer cell lines were screened for the presence of the GIRK1 potassium channel by RT-PCR analysis. The ER(+) cell lines MCF-7, MDA-MB-361 and ZR-75-1 and the ER(-) cell line MDA-MB-453 expressed mRNA for the GIRK1 channel (Figure 1). The ER(-) cell lines MDA-MB-468 and MDA-MB-435S did not express GIRK1 (Figure 1). GIRK1 is also not expressed in the normal breast epithelial cell line MCF 10A (data not shown). The PCR product from the MDA-MB-453 cell line was sequenced to verify the integrity of the PCR process and found to be homologous to the published sequence (data not shown). The PCR primers were designed to span exon 1 and 2 of GIRK1 [29]. In addition PCR amplification of negative control reactions (without the reverse transcriptase enzyme, data not shown) indicated that this was actually representative of mRNA expression and there was no contaminating genomic DNA. Since the GIRK1 potassium channels work as heterotetramers, we needed to determine which other GIRK channels were expressed in these breast cancer cell lines. As determined by RT-PCR, GIRK4 was expressed in all six breast cancer cell lines (Figure 2), and GIRK2 was expressed in four of the six cell lines. GIRK2 was not expressed in ZR-75-1 or MDA-MB-435S cell lines (Figure 2).

**Figure 3:**
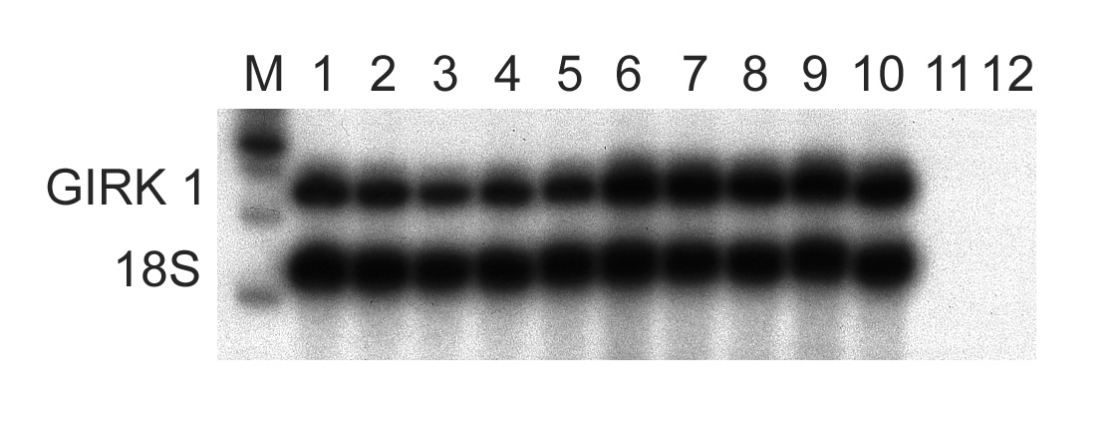
**Comparison of GIRK1 mRNA expression levels by relative competitive RT-PCR in MDA-MB-453 cells treated with propranolol constantly for 6 days. **Propranolol (1 μM) was added daily for six days. cDNA was amplified by PCR using GIRK1 primers and 18S primers/competimers. Lanes 1–5) untreated control; Lanes 6–10) propranolol treated cells; Lane 11) untreated control, RT reaction without MMLV; Lane 12, MDA-MB453 treated with propranolol, RT reaction without MMLV. Densitometry values were determined using the phosphoimager. Densitometry values of the bands for Girk 1 were normalized by the densitometry values of the bands for the 18S primers/competimers. Normalized values for control were 0.5494 ± 0.0285 (SD); and normalized values for propranolol treated were 0.9028 ± 0.0348 (SD), p < 0.0001. The bands were consistent with the expected sizes, 441 bp for the GIRK 1 primers and 324 bp for the 18S primers/competimers.

To determine if GIRK channels are functionally linked with β-adrenergic receptors in breast cancer cells expressing this ion channel, we decided to investigate the ER(-) cell line MDA-MB-453. This ER (-) cell line, which was the only ER(-) cell line tested that expressed GIRK1, was used for further experiments due to the fact that ER(-) breast cancers have a poorer prognosis than ER(+) cancers [2,3]. In addition, previous research in our laboratories indicated that this cell line expressed the β_2 _adrenergic receptor but not the β_1 _receptor [24]. MDA-MB-453 were continuously exposed to the beta-blocker propranolol (1 μM) for 6 days. Previous results from our laboratories indicated that maximal inhibition of breast cancer cell proliferation was at 1 μM propranolol [24]. Using relative RT-PCR, we saw a significant increase in GIRK1 channel mRNA expression (1.6 fold, Figure 3) after 6 days of continuous exposure to propranolol (p < 0.0001 by t-test). In these experiments, propranolol was added fresh each day. We also saw a significant decrease (1.5 fold) in β_2_-adrenergic receptor mRNA (p < 0.0079 by t-test) (data not shown).

**Figure 4:**
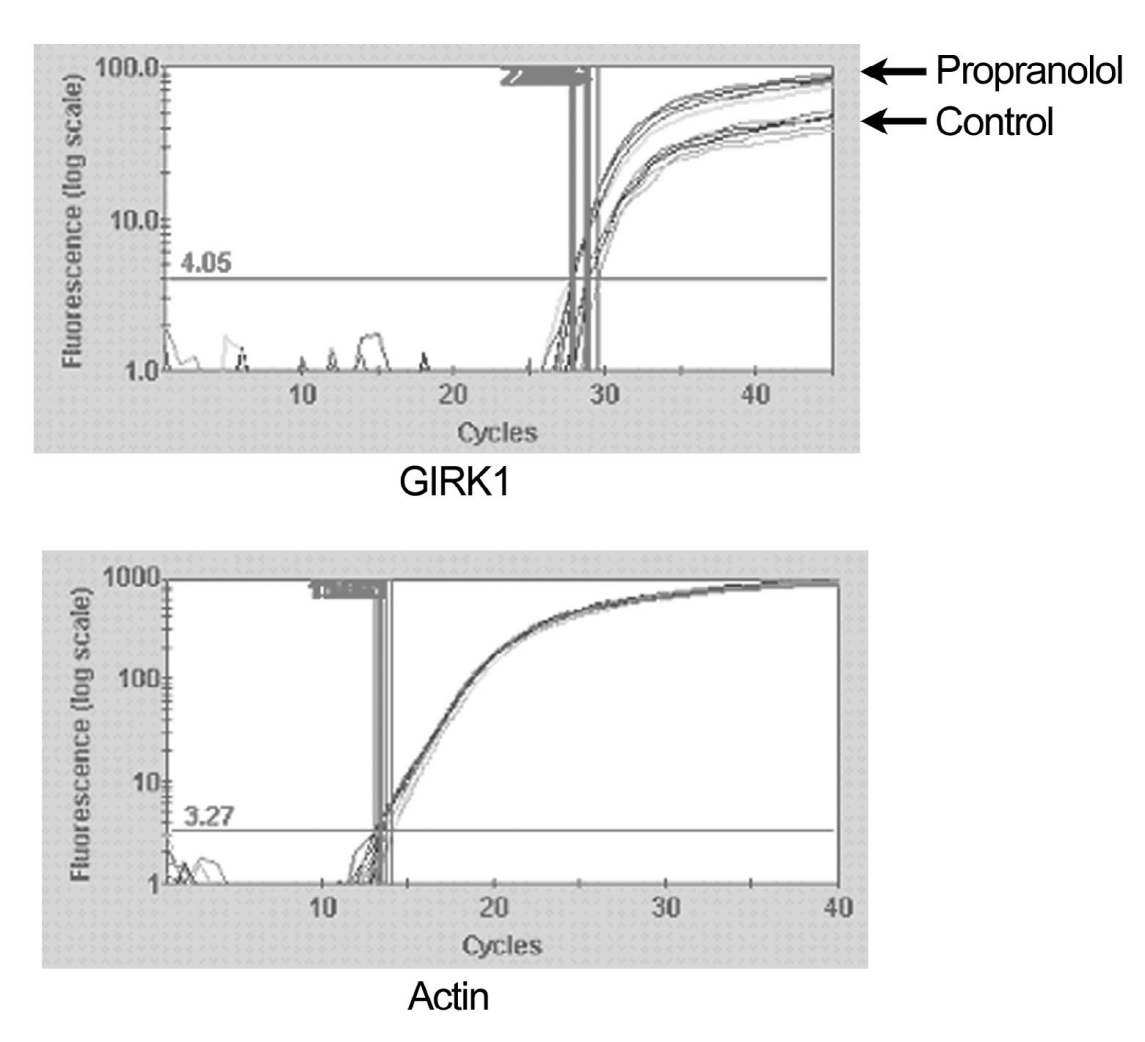
**Comparison of GIRK1 mRNA expression levels by real time RT-PCR in MDA-MB-453 cells treated with propranolol constantly for 6 days. **Propranolol (1 μM) was added daily for six days. Real time RT-PCR graphs of mRNA expression levels of GIRK1 and beta-actin. The graphs are from a Cepheid Smart Cycler using the same cDNA samples as used in Figure 2. N = 5 for each.

**Figure 5:**
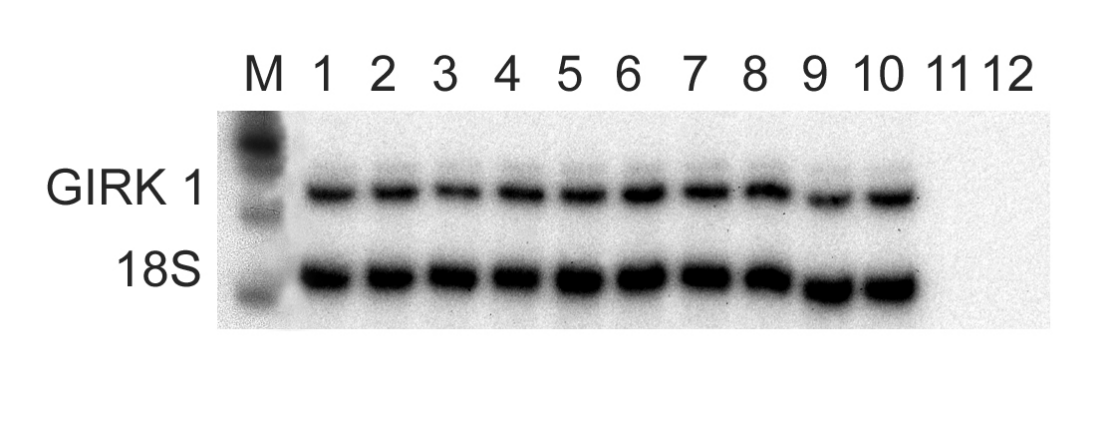
**Comparison of GIRK1 mRNA expression levels by relative competitive RT-PCR in MDA-MB-453 cells treated with propranolol for 30 minutes daily for 7 days. **Propranolol (1 μM) was added daily for seven days and then removed after 30 minutes. cDNA was amplified by PCR using GIRK1 primers and 18S primers/competimers. Lanes 1–5) untreated control; Lanes 6–10) propranolol treated cells; Lane 11) untreated control, RT reaction without MMLV; Lane 12, MDA-MB453 treated with propranolol, RT reaction without MMLV. Densitometry values were determined using the phosphoimager. Densitometry Girk 1/ Densitometry 18S-control-0.6055 ± 0.0685 (SD); propranolol treated 0.5636 ± 0.0611 (SD). The bands were consistent with the expected sizes, 441 bp for the GIRK 1 primers and 324 bp for the 18S primers/competimers.

Using the same cDNA samples, we performed a real-time RT-PCR assay using GIRK1 primers designed for real-time PCR and a TaqMan probe. We also saw a significant increase of GIRK1 mRNA using this method (Figure 4) and no change in the control, beta-actin values. Threshold values (C_T_) were calculated for each sample, which will be lower for samples with more mRNA expression. C_T _values for GIRK 1 expression were significantly (p < 0.001 by t-test) lower for propranolol treated cells (27.808 ± 0.107) (SD) as compared to control MDA-MB-453 cells (28.964 ± 0.338) (SD). Actin C_T _values were unchanged between control (13.666 ± 0.286) (SD) and propranolol treated cells (13.404 ± 0.427) (SD). The exposure to propranolol caused a slight decrease in GIRK2 mRNA expression (p < 0.04 by t-test) in the treated cells, opposite the result we found for GIRK1. Control (C_T _cycle values-31.35 ± 0.73) and propranolol (C_T _cycle values-32.24 ± 0.38). GIRK4 expression levels were unchanged between control and propranolol treated cells, indicated by real time PCR. Control (C_T _cycle values-33.0 ± 2.3) and propranolol treated (C_T _cycle values-31.7 ± 0.38). By contrast, MDA-MB-453 cells treated for 30 minutes daily for 7 days with 1 μM propranolol did not show changes in GIRK1 mRNA expression levels (Figure 5). No significant differences in GIRK1 mRNA expression were seen when MDA-MB-453 cells were exposed to 1 μM of either propranolol or the broad spectrum β-adrenergic agonist isoproterenol for 24 hours (data not shown).

**Figure 6:**
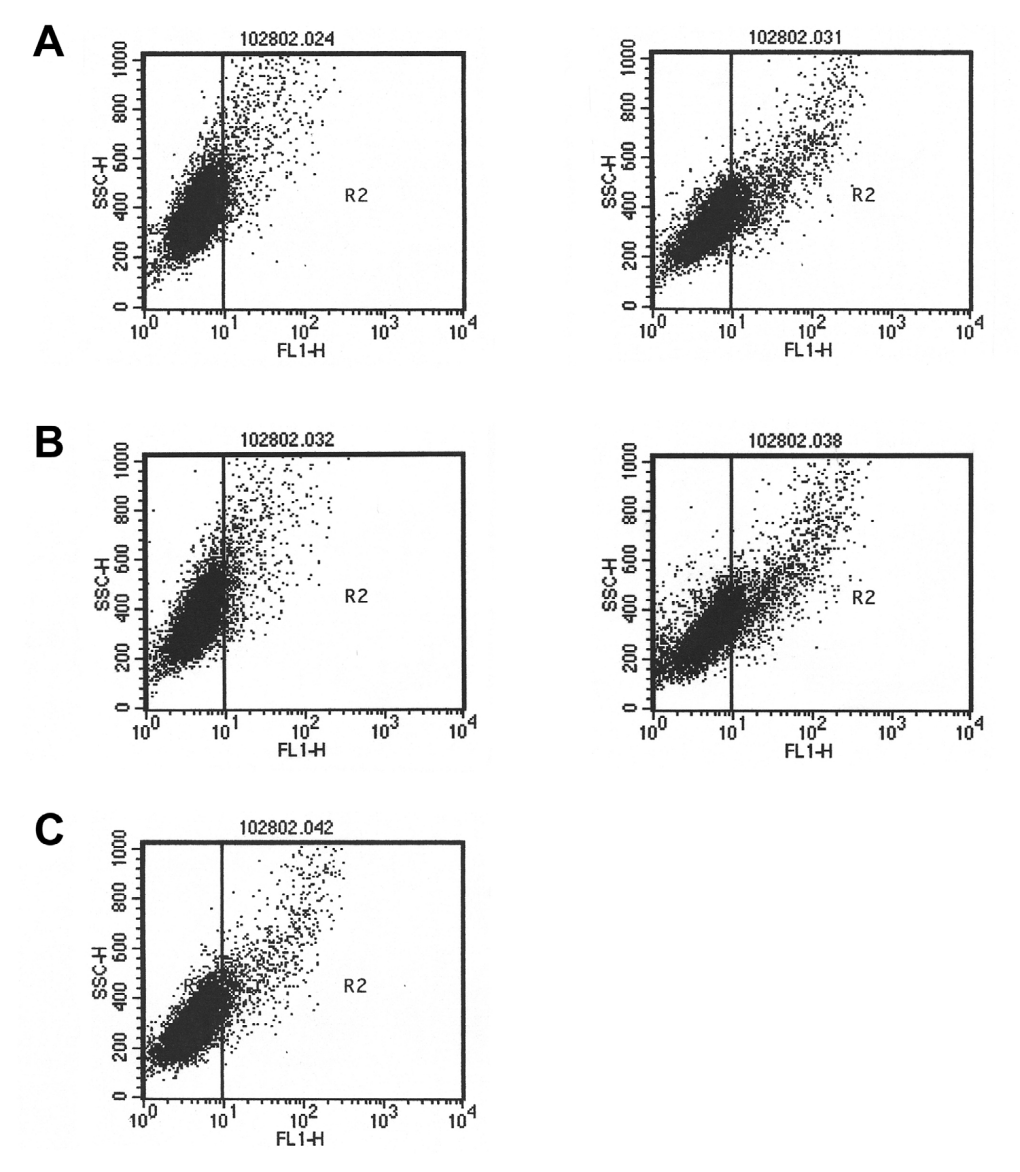
**Flow cytometry graphs showing potassium flux in MDA-MB-453 cells by the β_2 _agonist formoterol hemifumarate. **A) Fluorescence in R2 (inside the cell) increased from 9.37% to 18.82% after 1 μM formoterol treatment. B) Fluorescence in R2 increased from 8.96% to 21.14% after formoterol treatment C) Fluorescence levels in R2 remained at control levels at 10.63% after addition of 50 μM clozapine along with formoterol.

Although the gene expression studies showed no effects at shorter time periods or when it was not in the media constantly, we wished to determine if other cellular function are affected at shorter time periods in MDA-MB-453 cells. Potassium flux into cells would be an important part of any cellular response involving GIRK channels. We determined inward potassium flux in MDA-MB-453 cells by flow cytometry. The negatively charged fluorescent dye bis-(1,3-dibutylbarbituric acid)trimethine oxonol (DiBaC_4_(3)) was added to MDA-MB-453 breast cancer cell line suspensions of 1 × 10^6 ^cells at a final concentration of 150 × 10^-9 ^M. Fluorescence intensity measurement after treatment of the cells was obtained from a FACS Vantage/SE Cell Sorter. An increase of dye fluorescence corresponds to membrane potential depolarization and K^+ ^flux. The β_2 _selective agonist, formoterol hemifumarate (1 μM), added to MDA-MB-453 cell suspensions at the same time as the fluorescent dye lead to a 2X increase of fluorescence inside the cells, indicating inward potassium movement (Figure 6A &6B). Dye alone added to cells had no effect (data not shown). The GIRK inhibitor clozapine [31] (50 μM) added just prior to dye and formoterol addition completely blocked the effect of the beta-adrenergic agonist formoterol, (Figure 6C) indicating that blockage of the GIRK channel inhibited potassium flux, and that effects of beta-adrenergic agents on this breast cancer cell line are indeed mediated by GIRK channels.

**Figure 7:**
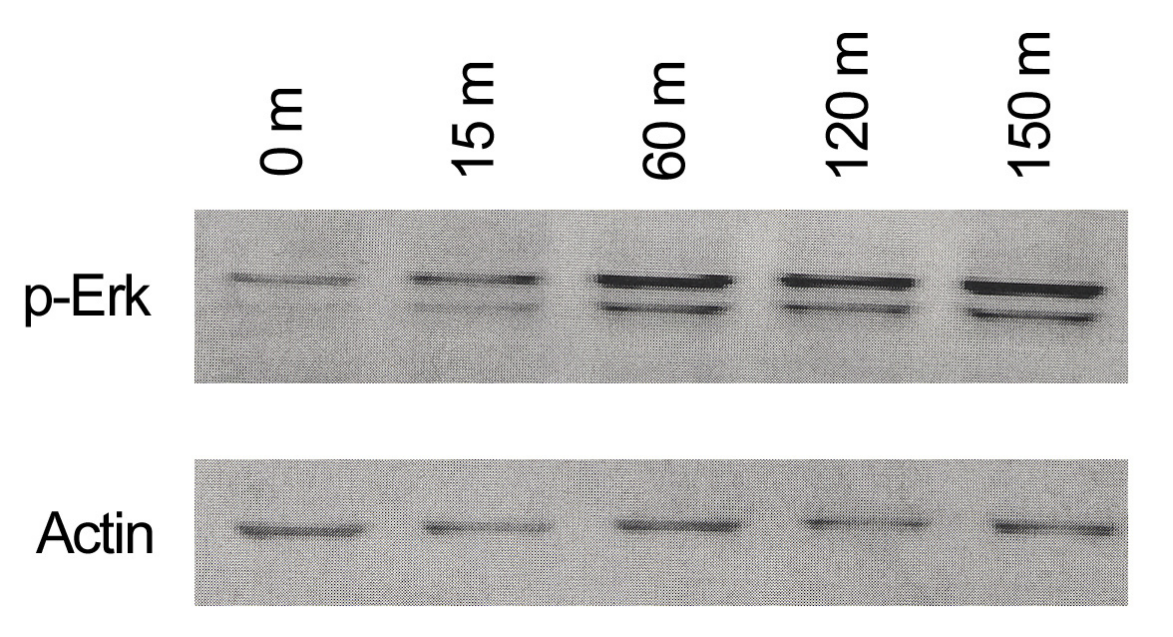
**Levels of ERK activation in MDA-MB-453 cells as assessed by western blot analysis. **Activation was determined after indicated times after exposure to 100 pM NNK using a phospho-specific antibody.

We also determined signaling events in MDA-MB-453 cells that are affected by beta-adrenergic agents. Increased activation of Erk 1/2 was seen in MDA-MB-453 cells after treatment with 100 pM NNK at times ranging from 15–150 minutes (Figure 7). The concentration of NNK used by us is within the range of systemic NNK concentrations found in smokers. In addition, an experiment in Patas monkeys [32] has shown blood levels of 1.6 pg/ml (7.72 × 10^-12 ^M) after exposure to a dose of tritiated NNK equivalent to the amount of NNK found in two packs of cigarettes. Stimulation of Erk 1/2 was also seen using 1 μM of the beta-adrenergic agonist formoterol, but only at 150 minutes (data not shown).

## Discussion

Our data demonstrate expression of the G-protein inwardly rectifying potassium channel 1 (GIRK1) in 67% of the breast cancer cell lines tested, with higher levels in ER(+) cell lines. Approximately 40% of primary human breast cancers were found to express GIRK1 and expression of GIRK1 was not found to be correlated with ER status [25]. These differences in our studies may be due to the subset of breast cancer cell lines tested. We also found that the normal breast epithelial cell line MCF 10A lacked GIRK1 expression (data not shown). GIRK1 cannot form functional channels by itself, other GIRK channels are needed [33]. All six breast cancer cell lines tested express either GIRK2 or GIRK4 indicating that functional GIRK potassium channels are possible in these breast cancer cell lines.

The majority of experiments in the present study were done with the ER(-) cell line MDA-MB-453 since it was the only ER(-) cell line tested that expressed GIRK1, and because ER(-) breast cancers have a poorer prognosis than ER(+) cancers [2,3]. We saw a significant increase in GIRK1 channel mRNA expression after 6 days of continuous exposure to propranolol in MDA-MB-453 cells. It is clear that at least six days of continuous exposure to the beta-blocker propranolol is necessary to effect gene expression. Gene expression of β_2_-adrenergic mRNA was decreased by the same treatment (data not shown). Addition of propranolol for 7 days for only 30 minutes daily had no effect on GIRK1 gene expression. Treatment for a shorter period of time (24 hours) also had no effect on GIRK1gene expression in our studies. The 6 day continuous exposure to propranolol caused a barely detectable decrease in GIRK2 mRNA expression and no change in GIRK4 mRNA expression levels. Longer treatment times may be necessary for gene expression changes in GIRK2 or GIRK4 similar to gene expression changes that are seen in GIRK1.

Although there were no short-term effects of beta-adrenergic agents on GIRK gene expression, we detected other cellular effects. The beta-adrenergic agonist formoterol hemifumarate stimulated potassium influx in MDA-MB-453 cells, and this influx was prevented by the GIRK channel inhibitor clozapine. NNK, a high affinity agonist for beta-adrenergic receptors [11] increased activation of Erk 1/2 in MDA-MB-453 breast cancer cells. Formoterol also increased activation of Erk 1/2, but to a lesser degree (data not shown). Previous studies indicated that the beta-adrenergic agonist isoproterenol stimulates growth [24]. GIRK currents have been shown to be increased in cells stimulated with the beta-adrenergic agonist isoproterenol in rat atrial myocytes transfected with β_1_or β_2 _receptors [27]. Heterologous facilitation of GIRK currents by β-adrenergic stimulation was also seen in rat cardiomyocytes [26]. Two polymorphisms in the β_2 _and β_3 _adrenergic receptors were found to be correlated with a decreased risk for breast cancer [34], suggesting an important role of this receptor family in the genesis of breast cancer. In previous work, we demonstrated mRNA expression by RT-PCR of the β_2 _adrenergic receptor in the six breast cancer cell lines used in this study, but expression of β_1 _in all the estrogen responsive cell lines but not in two ER(-) cell lines (MDA-MB-435S and MDA-MB-453) [24]. Further studies are needed to determine how GIRK1(+) and ER(-) breast cancers are regulated and if GIRK channel agonists and antagonists have effect on proliferation in breast cancer. It also remains to be determined if this same regulation is present in GIRK1(+) and ER(+) breast cancer malignancies. This is of particular importance since a recent report indicated that 17-β-estradiol can modulate GIRK channel activation in the brain [35]. Future studies are also needed to determine if GIRK3 is involved in breast cancer. However, we think this unlikely because one of the functions of GIRK3 is to inhibit plasma membrane expression of other GIRK subunits [36].

## Conclusions

All six breast cancer cell lines tested express either GIRK2 or GIRK4 indicating that functional GIRK potassium channels are possible in these breast cancer cell lines. This is the first report that implicates β-adrenergic receptors and G-protein inwardly rectifying potassium channels 1 (GIRK1) in the regulation of human breast cancer cells and suggests a potential role of the tobacco nitrosamine NNK in breast cancers expressing these regulatory pathways. Beta-adrenergic antagonists have both long term effects on gene expression and beta-adrenergic agonists have short term effect on potassium flux and cellular signaling pathways.

## Competing interests

The author(s) declare they have no competing interests.

## Authors' contributions

HP carried out the majority of experiments and participated in the design of the study, and helped draft the manuscript. QY carried out the western blots. YC was involved in relative competitive RT-PCR studies. HS conceived of the study and helped draft the manuscript.

## Pre-publication history

The pre-publication history for this paper can be accessed here:
